# Supplementary material for: Bacterial Cell Display as a Robust and Versatile Platform for Engineering Low‐Affinity Ligands and Enzymes
Source: Chembiochem. 2020 Jun 29;21(19):2844–53. doi: 10.1002/cbic.202000203 (PMC7586821; doi:10.1002/cbic.202000203)
Supplement: Supplementary file 1 — Supplementary [file CBIC-21-2844-s001.pdf]

ChemBioChem

Supporting Information

# Bacterial Cell Display as a Robust and Versatile Platform for Engineering Low-Affinity Ligands and Enzymes**

Eszter Csibra, Marleen Renders, and Vitor B. Pinheiro*

## Author Contributions

E.C. Data curation:Lead; Formal analysis:Lead; Investigation:Lead; Methodology:Equal; Writing - Original Draft:Equal; Writing - Review & Editing:Equal
M.R. Investigation:Supporting; Methodology:Supporting; Writing - Review & Editing:Supporting
V.P. Conceptualization:Lead; Data curation:Supporting; Formal analysis:Supporting; Funding acquisition:Lead; Methodology:Equal; Project administration:Lead; Supervision:Lead; Writing - Original Draft:Lead; Writing - Review & Editing:Lead

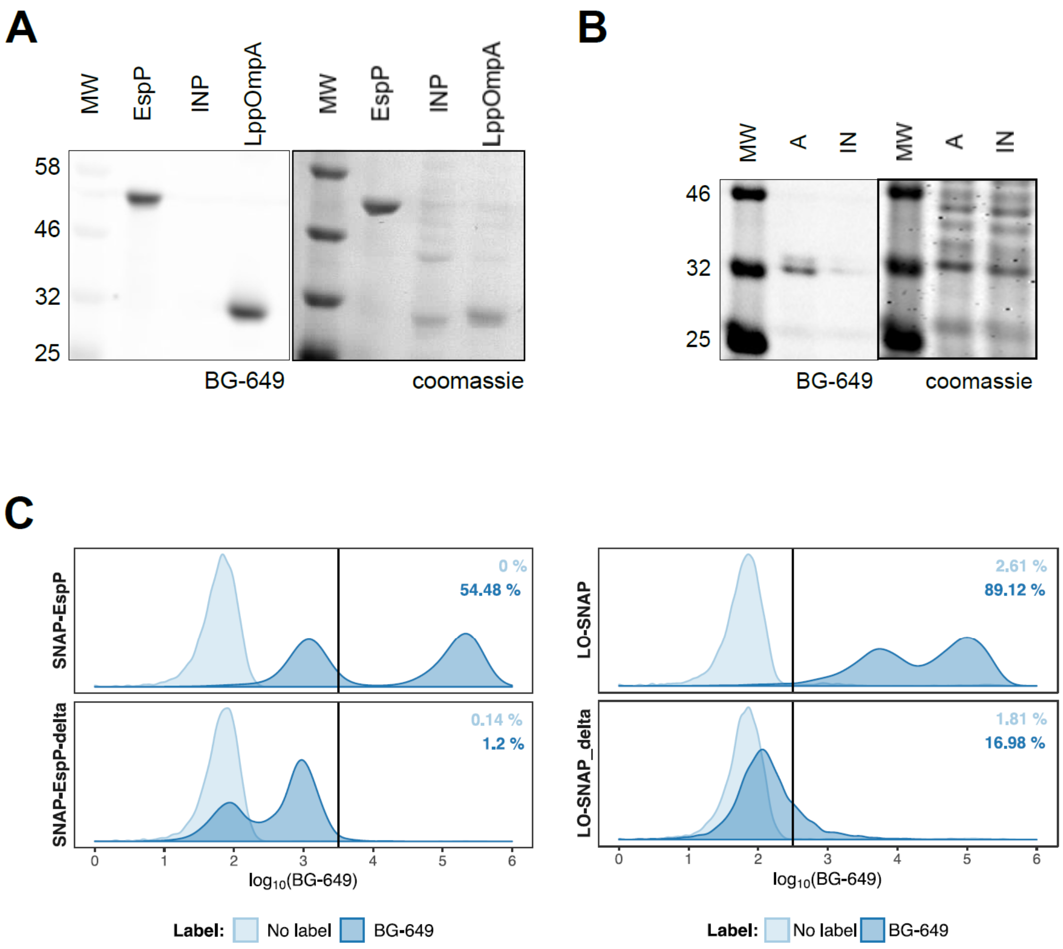


**SF1. Cell surface SNAP expression and activity analysed by SDS-PAGE and flow cytometry. A.** SNAP expression (Coomassie panel) and activity (BG-649 panel) by display construct, stained as in Fig1b, showing insoluble protein fractions separated by SDS-PAGE and imaged by fluorimetry (Typhoon imager). Next to the EspP and LppOmpA systems, the ice nucleation protein (INP) system was also compared here. INP constructs structure resembled LppOmpA constructs in that the protein of interest is displayed on the C-terminal end of the fusion. **B.** SNAP expression (Coomassie panel) and activity (BG-649 panel) in active (SNAP-EspP) and inactive (SNAP-delta-EspP, missing residues 83-100) variants. **C.** SNAP activity as analysed by flow cytometry. Cells were incubated with BG-649, washed in PBS and analysed using an Attune NxT cytometer, RL1-H channel (10,000 cells/sample). Histograms show cell signal in the RL1 channel, while annotations (%) indicate the proportion of cells considered positive (that score over the indicated gate threshold value) for BG-649 binding. The gate acts as an indicator of the proportion cells

that would be sorted by FACS in a selection experiment based on fluorescence rather than biotin/bead binding.

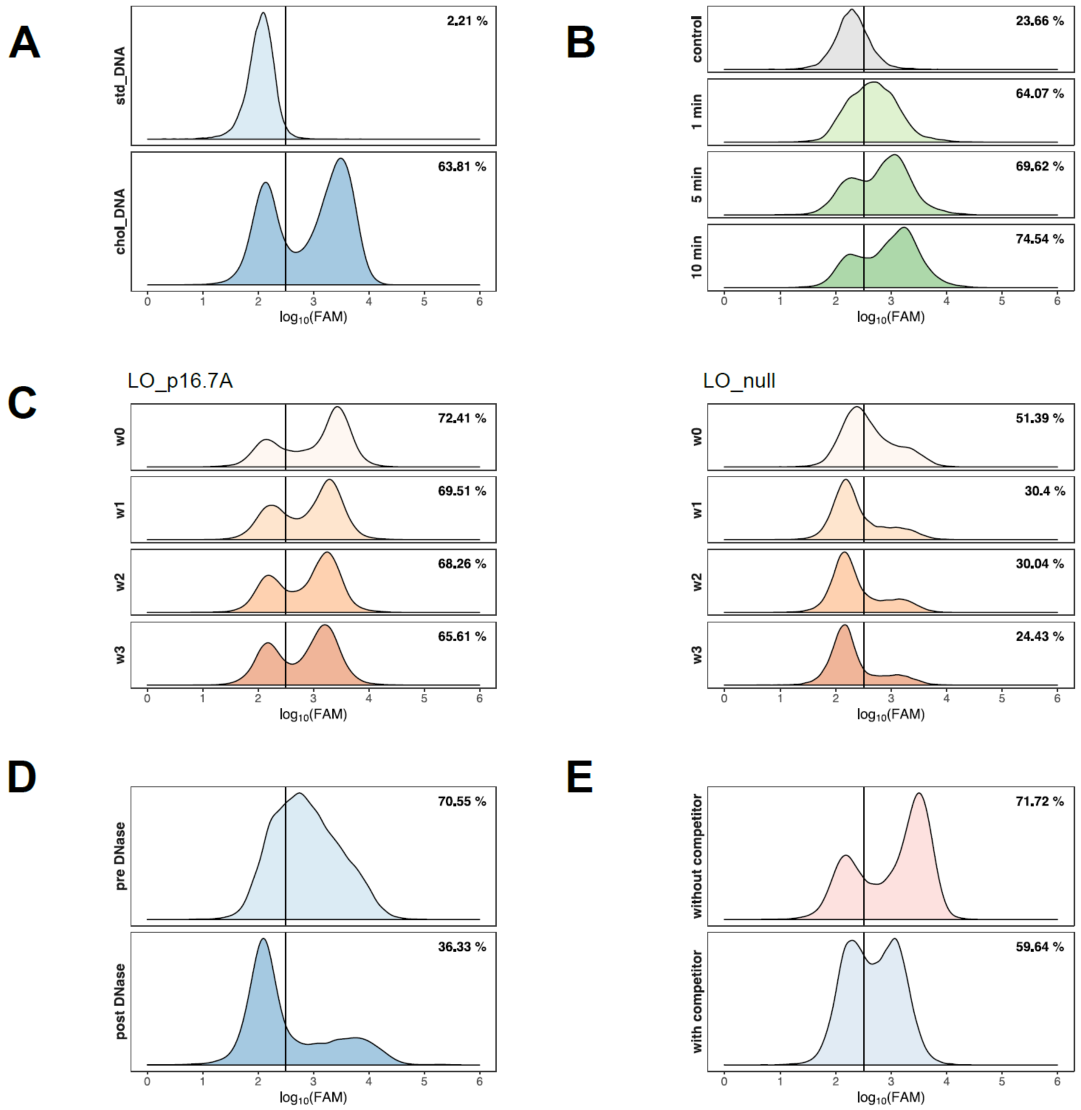


**SF2. Characterisation of cholesterol-linked DNA binding to cells with displayed p16.7 protein. A.** Comparison of FAM signal detectable on cells (pLO16A) mixed with cholesterol-labelled DNA (dsDNA: EC98a/EC98b_chol) versus unlabelled DNA (EC98a/EC98b). **B.** Timecourse of DNA binding to cells. Cells (pLO16A) were mock labelled (control) or incubated with DNA (EC98b_chol) for the indicated time (30$^{o}$C, with shaking), before being washed in PBS and analysed. **C.** Analysis of the stability of cholesterol-DNA bound to cells. Cells (pLO16A, *left*; pLO16A_deltaC, *right*) were incubated with DNA (EC98b_chol, 30 min at 30$^{o}$C with shaking), before being diluted for analysis (w0) or washed in PBS, recovered by pelleting and then diluted (w1-3) the indicated number of times. **D.** Analysis of DNA accessibility to the surrounding media.

Cells (pLO16A) were incubated with DNA (EC98b_chol, 30 min at 30$^{o}$C with shaking), washed, and subjected to DNase treatment (Turbo DNase, 37$^{o}$C, 30 min), before washing and dilution for analysis. **E.** Analysis of the process of DNA:cell binding. Cells (pLO16A) were incubated with DNA (EC98b_chol, 30 min at 30$^{o}$C with shaking), without (*top*) or with (*bottom*) the addition of excess competitor DNA (EC98b, without the cholesterol moiety) to ascertain whether the binding was driven by DNA:DNAbp interactions or cholesterol:membrane interactions. (The decrease in FAM signal suggests that the competitor, despite not having a cholesterol moiety, interferes with cholestol-DNA:cell binding, suggesting that binding of cholesterol-DNA is DNA:DNAbp driven).

Histograms show cell signal in the BL1-H channel, while annotations (%) indicate the proportion of cells considered positive (that score over the indicated gate threshold value) for DNA binding. Overlapping traces show data from triplicate samples; samples shown in singlet form are representative of multiple experiments.

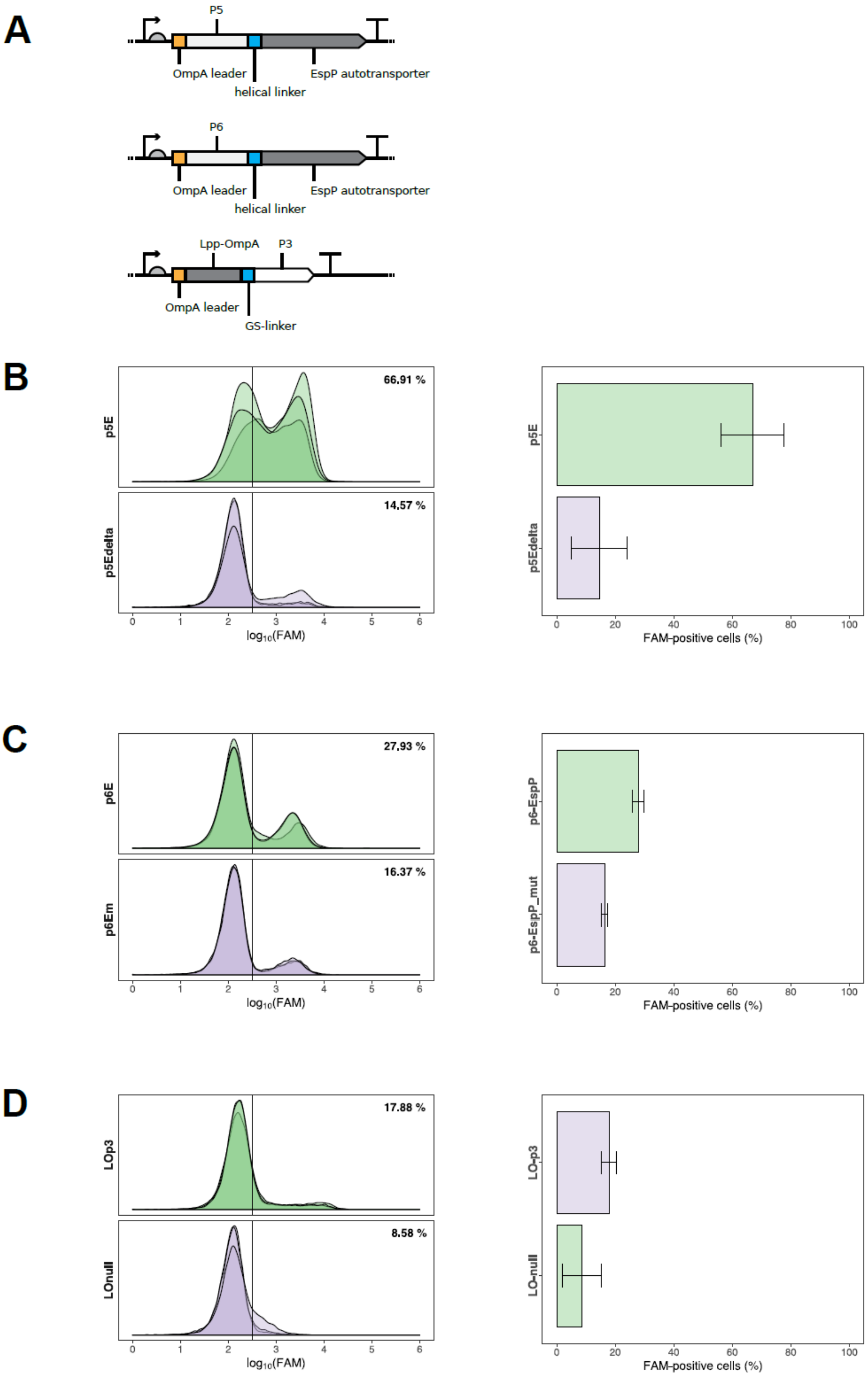


**SF3. Cell surface display of DNA binding proteins from phi29. A.** Constructs used for

DNA-binding protein display, showing leader sequence (orange), membrane protein (grey), linker (blue), DNA-binding protein (yellow). **B-D.** Assessmengt of DNA-binding by cells, using active and inactive variants of each protein, showing signal from FAM-labelled DNA on cells by flow cytometry (*left*) and the proportion of cells scoring as FAM-positive (*right*). **B.** p5 (ssDNA binding protein) display using EspP, with p5-delta (missing residues 44-61) as the inactive control. **C.** p6 (dsDNA binding protein) display using EspP, with p6-mutant (K3A, R7A, K11A, T12A) as the inactive control. **D.** p3 (ss/dsDNA binding protein) display using LppOmpA, with LO-null as the inactive control.

Cells were induced for expression of the indicated cell surface displayed DNA binding protein, washed, incubated with ssDNA (EC98a) or dsDNA (EC98b_chol), washed and analysed by flow cytometry (Attune NxT, 10,000 cells/sample). Histograms show cell signal in the BL1-H channel, while annotations (%) indicate the proportion of cells considered positive (that score over the indicated gate threshold value) for DNA binding. Overlapping traces show data from triplicate samples. Error bars indicate standard deviation.

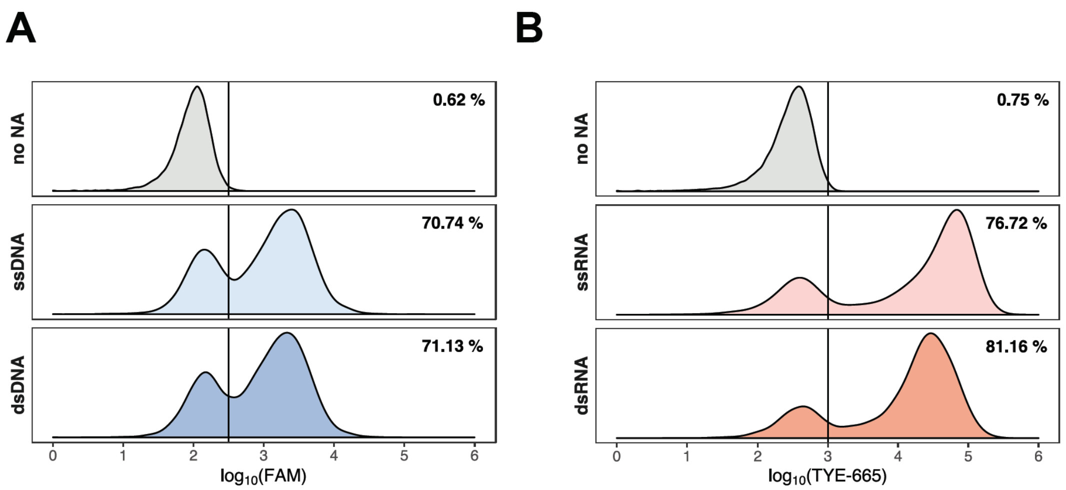


**SF4. Displayed p16.7 binds RNA.** Cells (pLO16A) were incubated with (**A.**) ss/dsDNA (EC98b_chol/EC98a, FAM) or (**B.**) ssRNA (EC184_rev_chol/EC184_ fwd, TYE-665), for 30 min at 30$^{o}$C with shaking, before being washed and analysed by flow cytometry. Histograms show cell signal in the BL1-H channel for FAM and RL1-H for TYE-665, while annotations (%) indicate the proportion of cells considered positive (that score over the indicated gate threshold value) for DNA binding. Samples are representative of multiple experiments.

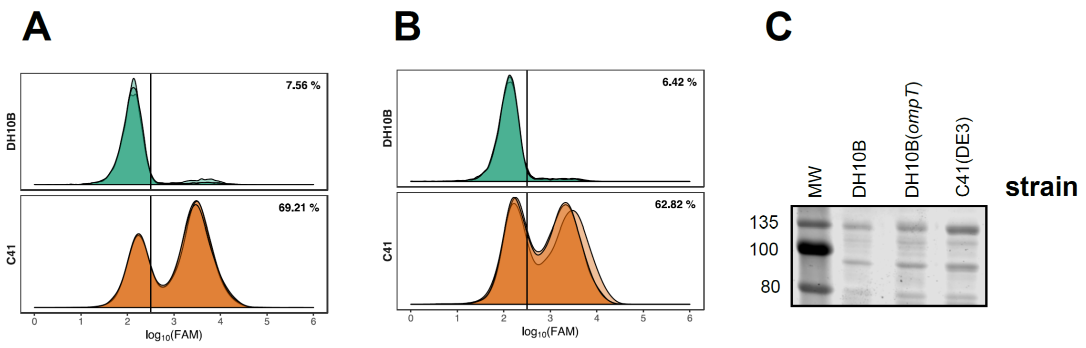


**SF5. Expression of cell surface polymerase.** Constructs for cell surface polymerase expression consisted of a fusion of the phi29 DNA polymerase to the EspP autotransporter (pP2-EspP, SI Table 1), the rest of the vector identical to pSNAP-EspP. The activity of P2-EspP for DNA binding was analysed by flow cytometry. DH10β and C41 strains were transformed with the construct, induced and mixed with cholesterol-linked ss- (**A**) and ds- (**B**) DNA before being washed and analysed by flow cytometry. **C.** Expression of P2-EspP by strain. DH10β and C41 strains (and a DH10β variant with an *ompT* knockout, a gift from Valérie Pezo and Philippe Marlière) were analysed for their expression of P2-EspP (102 kDa) by SDS-PAGE

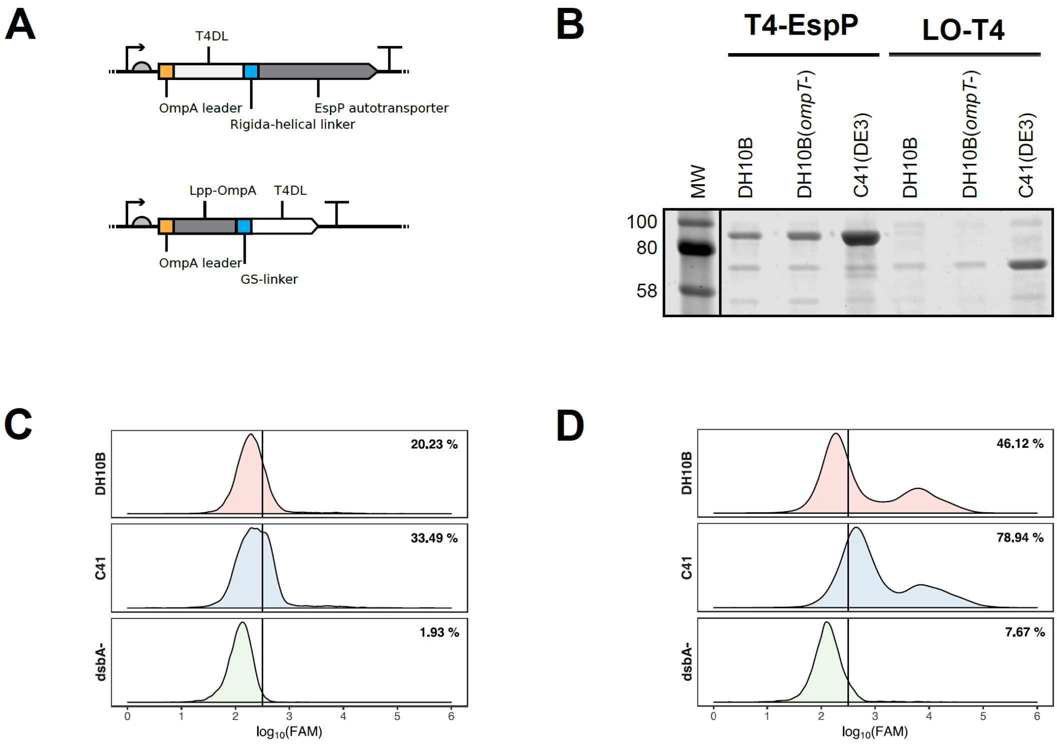


**SF6. Cell surface display of T4 DNA ligase. A.** Ligase display constructs, showing leader sequence (orange), ligase variant (yellow), linker (blue) and membrane protein (grey). **B.** Expression of cell surface ligase by construct and strain. DH10β and C41 strains (and a DH10β variant with an *ompT* knockout) were analysed for their expression of T4-EspP (91 kDa) and LO-T4 (69 kDa) by SDS-PAGE. **C-D.** Activity of T4DL for DNA binding by construct and strain. DH10β, C41 and BW25113 *dsbA* strains were transformed with the construct, induced and mixed with cholesterol-linked ss- (**C**) and ds- (**D**) DNA before being washed and analysed by flow cytometry.

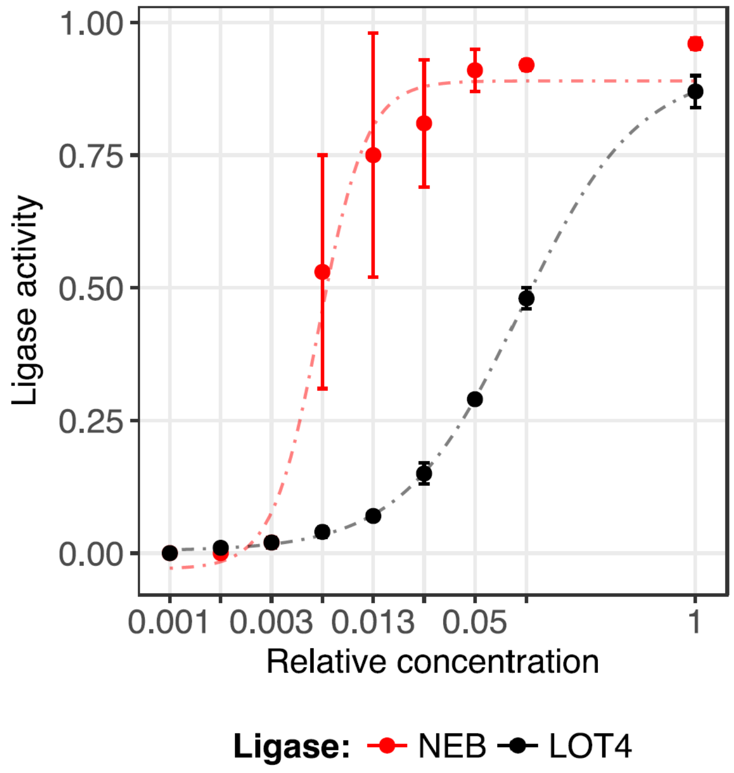


**SF7. Quantification of T4DL activity compared to NEB T4DL.** Dilution series of NEB T4 DNA ligase (in red) and T4DL-displaying cells (LOT4, C41, in black) were constructed and mixed with pre-annealed splint ligation substrates MR9/10-F1/11-54PS (as in Fig. 4b) and NEB T4 DNA ligase buffer (1X). Ligation products (37$^{o}$C, 1h; using only the DNA-containing supernatant for the cell samples) were separated by ureaPAGE, imaged (Typhoon imager) and quantified by densitometry (ImageJ). Ligase activity was calculated as the band intensities of: ligated product / (ligated + not ligated species), normalised to 1 for the undiluted NEB T4 DNA ligase. Data is presented as the mean of duplicate experiments (error bars = standard deviation) along with curve-fitting to a Hill function (dashed lines).

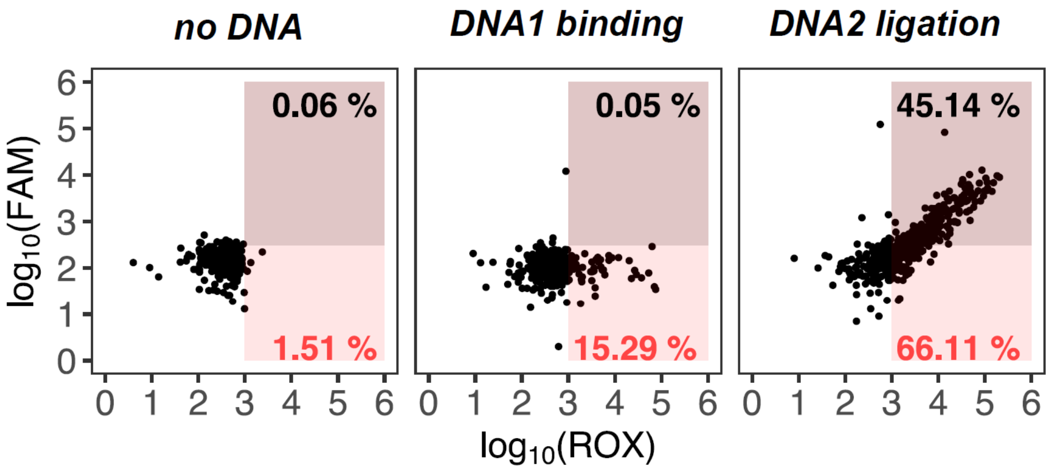


**SF8. Flow cytometric analysis of the steps in ligase selection using fluorophore-linked DNA.** Ligase activity using cell surface-linked substrates. Ligation substrates were as described in Fig 4c. Ligation activity was analysed by flow cytometry for T4DL displaying cells (LOT4, C41) before the addition of DNA (*left*), after DNA1 binding(*centre*), and after DNA2 ligation (*right*). Plots show a sample of 1000 cells for clarity (10,000 collected), and indicate the proportion of cells considered positive for (i) DNA attachment (x axis, ROX label), and (ii) Ligation (y axis, FAM label).

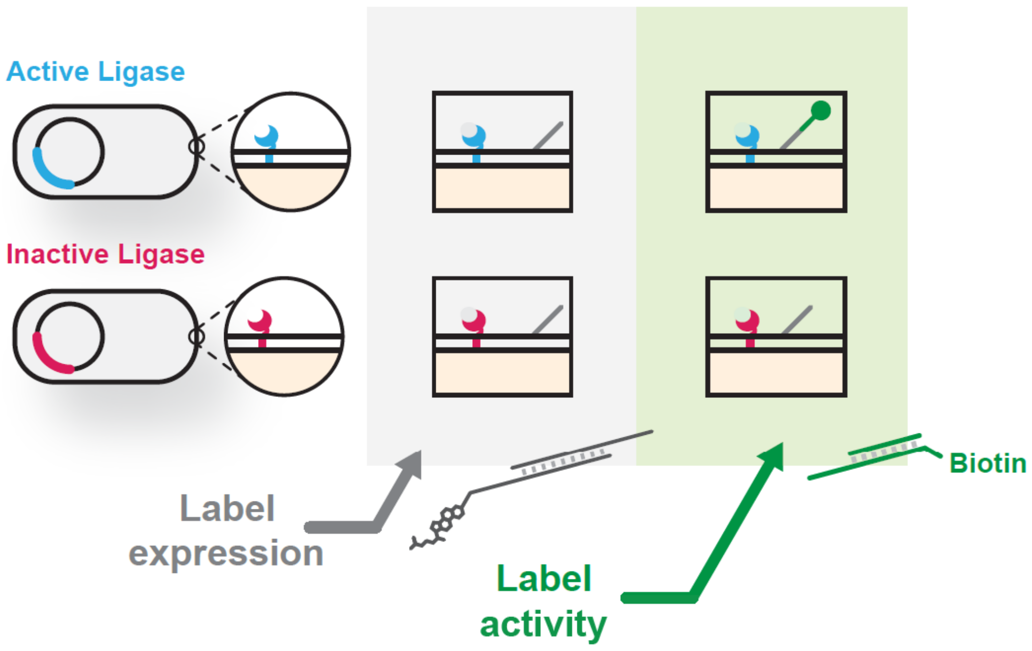


**SF9. Ligase selection method using biotin-linked DNA.** Active and inactive displayed ligases were tested in model selection experiments as follows. Cells were subjected to DNA1 binding (grey background), washing to remove nons-transient interactors, followed by DNA2 ligation (green background). Note that DNA2 here is linked to biotin, not FAM. Cells were washed again, and biotin-labelled cells were captured by binding to streptavidin-coated beads as described in the Methods.

## Supplementary Table Legends

### Table S1. Oligonucleotides for cloning.

Table S1 details the primers used for DNA amplification for each construct, the vectors used for the fragments and the method used for assembly.

### Table S2. Oligonucleotides for DNA binding, ligase assays and ligase selection

Table S2 details primers used for model selection PCRs as well as DNA binding and ligase assays on the cell surface. DNA modifications are annotated using the same codes used by IDT (https://www.idtdna.com/pages/products/custom-dna-rna/oligo-modifications).

### Table S3. Plasmids.

### Table S4. Strains.

**Table S5. Gene sequences.** Novel gene sequences that are not available from other sources are provided. LppOmpA and p16.7 were ordered as gblocks, whereas the INP sequence provided is the relevant fragment from plasmid pUC57-INPnc-H.

**Table S1.** **Oligonucleotides for cloning**

| To make plasmid | Vector fragment from: | Insert fragment from | Primer ## | name | EC Primer nam | Sequence | Assembly method |
|---|---|---|---|---|---|---|---|
| **SNAP series** | *pSNAP-EspP2: Part 1. Assembled from pNGAL97, oligos as the linker, and SNAP ORF by Type IIS. Part 2: iPCR mutagenesis w EC12fwd/rev, blunt ligation, to bring it in frame.* | | | | | | |
| **pSNAP-EspP** | pNGAL97 | | 1 | _vecfwd | EC1fwd | AAACACCTGCATCAGCAAACAAGGAAGCAACC | Type IIS |
| | pNGAL97 | | 2 | _vecrev | EC1rev | TAACACCTGCATATGGCCTGCGCTACGGTAACG | |
| | | oligos | 3 | _insfwd | EC5a | CTGGCGGAAGCAGCGGCAAAAGAAGCAGCGGCCAAAGAAGCAGCGGCAAAAGAAGCAGCAGCGAAAGCGGCAGCG | |
| | | oligos | 4 | _insrev | EC5b3 | TTGCCGCTGCCGCTTTCGCTGCTGCTTCTTTTGCCGCTGCTTCTTTGGCCGCTGCTTCTTTTGCCGCTGCTTCCG | |
| | | pSNAP-tag(T7_2) | 5 | _ins2fwd | EC4fwd | AAACACCTGCATTAGGCCATGGACAAAGATTGCGA | |
| | | pSNAP-tag(T7_2) | 6 | _ins2rev | EC4rev | TAACACCTGCATATCCAGCCCAGACCCGGTTTAC | |
| **pSNAP-EspP2** | pSNAP-EspP | n/a | 7 | _vecfwd | EC12fwd | aCTGGCGGAAGCAGCG | Blunt ligation |
| | pSNAP-EspP | n/a | 8 | _vecrev | EC12rev | CCCAGACCCGGTTTAC | |
| **pSNAP_Esp2_delta** | pSNAP-Esp2 | n/a | 9 | _vecfwd | EC38fwd | AAAcacctgcATCAgaaaCTGCTGAAAGTGGTTAAATTTGG | Type IIS |
| | pSNAP-Esp2 | n/a | 10 | _vecrev | EC38rev | TAAcacctgcATATtttcGGAACCGGAAATTCTTCAATCG | |
| **INP and LO systems** | *LO-SNAP was created from INP-SNAP. Step1: assembly of pINP-SNAP. Step2: Replacement of INP with LO segment.* | | | | | | |
| pLO_SNAP (step1: pINP-SNAP) | pNGAL97 | | 11 | _vecfwd | EC16fwd | AAAcacctgcATCAtaagCTTGACCTGTGAAGTGAAAAATG | Type IIS |
| | pNGAL97 | | 12 | _vecrev | EC16rev | TAAcacctgcATATttttTGCCCTCGTTATCTAGATTTTTG | |
| | | pUC57-INPnc-H | 13 | _insfwd | EC17fwd | AAAggtctcCaaaaATGACTCTCGACAAGGCG | |
| | | pUC57-INPnc-H | 14 | _insrev | EC17rev | TAAggtctcActttGcagctgcTTCTttagctgcagcttcc | |
| | | pSNAP-tag(T7_2) | 15 | _ins2fwd | EC18fwd | AAAcacctgcATCAaaagATGGACAAAGATTGCGAAATG | |
| | | pSNAP-tag(T7_2) | 16 | _ins2rev | EC18rev | TAAcacctgcATATcttaTCCCAGACCCGGTTTACC | |
| pLO_SNAP (step2) | pINP-SNAP | | 17 | _vecfwd | VP11F2 | aaaggtctcaatcaATGGACAAAGATTGCGAAATG | Type IIS |
| | pINP-SNAP | | 18 | _vecrev | VP11R | aaaggtctcaatgaGGTCTGCAAATTCTGCG | |
| | | LppOmpA gblock | 19 | _insfwd | VP13fwd | CCCAATCTAGAAAGGAGATATAC | |
| | | LppOmpA gblock | 20 | _insrev | VP13rev | aaaggtctcatgatccggtcgc | |
| pLO_SNAP_delta | - | - | - | - | - | *As for pSNAP_EspP2_delta* | - |
| **A:I series and null mutants** | *Construction of plasmids with active.inactive model selection island barcodes used same sets of primers and Gibson assembly. Null variants of the display constructs were used as controls.* | | | | | | |
| [Plasmid]_amsi | [various] | | - | _vecfwd | EC156fwd | CTATCAGCAGCTGGCGTAACTCACGGATCTCCACGC | Gibson |
| | [various] | | - | _vecrev | EC156rev | ATCAGCGGTTCCTTAGCTTAGACGCAGTAGCGGTAAAC | |
| | | pSNAP-Esp2 | 21 | _insfwd | EC157fwd | TAAGCTAAGGAACCGCTGATGCAGG | |
| | | pSNAP-Esp2 | 22 | _insrev | EC157rev | GTTACGCCAGCTGCTGATAGC | |
| [Plasmid]_imsi | [various] | | - | _vecfwd | EC156fwd | - | Gibson |
| | [various] | | - | _vecrev | EC156rev | - | |
| | | pSNAP_Esp2_delta | - | _insfwd | EC157fwd | - | |
| | | pSNAP_Esp2_delta | - | _insrev | EC157rev | - | |
| pEspP_null | pSNAP_EspP2 | | 23 | _vecfwd | EC150fwd | TCGTTACCGTAGCGCAGGCCCTGGCGGAAGCAGCG | Gibson |
| | pSNAP_EspP2 | | 24 | _vecrev | EC150rev | GGCCTGCGCTACGG | |
| pLO_null | pLO_SNAP | | 25 | _vecfwd | EC151fwd | cggaaattgcgaccggatcataagCTTGACCTGTGAAGTGAAAAATGG | Gibson |
| | pLO_SNAP | | 26 | _vecrev | EC151rev | tgatccggtcgcaatttccg | |
| **p16.7** | *p16.7A was assembled into pET23d+ vector, the transmembrane domain was removed (p16.7A), and this was inserted into the pLO vector. A cysteine was inserted into the linker for visualisation, and this was used to produced the mutants.* | | | | | | |
| pET23d_p16.7 | pET23d+ | | 27 | _vecfwd | EC72fwd2 | CAGGCATGCAAGCTTGC | SPLiCE |
| | pET23d+ | | 28 | _vecrev | EC72rev2 | CAGGTCGACTCTAGAGGATCC | |
| | | p16.7 gblock (used w/o PCR) | - | - | - | - | |
| pET23d_p16.7A | pET23d_p16.7A | | 29 | _vecfwd | EC77fwd | AAAcacctgcATTAcgaaACAACAAAAAGAAACAGGAAGC | Type IIS |
| | pET23d_p16.7A | | 30 | _vecrev | EC77rev | AAAcacctgcATATttcgCATGGTATATCTCCTTCTTAAAGTTAAACAAAATTATTTC | |
| pLO16A (step1, intermediate) | pLO_SNAP | | 31 | _vecfwd | EC81fwd | AAAggtctcAtaagCTTGACCTGTGAAGTGAAAAATG | Type IIS |
| | pLO_SNAP | | 32 | _vecrev | EC81rev | TAAggtctcAtgatccggtcgcaatttcc | |
| | | pET23d_p16.7A | 33 | _insfwd | EC82fwd | AAAcacctgcATATatcaCGAAACAACAAAAAGAAACAGGAAGC | |
| | | pET23d_p16.7A | 34 | _insrev | EC82rev | TAAcacctgcATTActtaTTTCAATGACCCCCGATATAGTTTTTTCTG | |
| pLO16A[cys] (step2, to use) | pLO16A | | 35 | _vecfwd | EC85fwd | AAAggtctcAtgcgGCAGCCGAAACAACAAAAAGAAACAGGAAGC | Type IIS |
| | pLO16A | | 36 | _vecrev | EC85rev | TAAggtctcTcgcatgatccggtcgcaatttcc | |
| pLO16A[cys]_delta | pLO16A[cys] | | 37 | _vecfwd | EC86fwd | AAAggtctcTctatATCGGGGGTCATTGAAAtaagC | Type IIS |
| | pLO16A[cys] | | 38 | _vecrev | EC86rev | TAAggtctcAatagCAAATCTTCGATGATGTCACTAGGAATG | |
| pLO16A[cys]_deltaC | pLO16A[cys] | | 39 | _vecfwd | EC68fwd | AAAggtctcAtaagCTTGACCTGTGAAGTGAAAAATGG | Type IIS |
| | pLO16A[cys] | | 40 | _vecrev | EC180rev | TAAggtctcActtaCTGATTCTCCAATTTCCAGTATGTCC | |
| **phi29 proteins** | *The phi29 proteins were inserted into the EspP or LO vectors by Type IIS.* | | | | | | |
| pLOp3 | pLO_SNAP | | 41 | _vecfwd | EC129fwd | AAAggtctcAtaagCTTGACCTGTGAAGTGAAAAATG | Type IIS |
| | pLO_SNAP | | 42 | _vecrev | EC129rev | TAAggtctcAagaccctccggtcgcaatttccg | |
| | | pT7_p3 | 43 | _insfwd | EC130fwd | AAAggtctcAgtctggaggttctATGGCGAGAAGTCCACG | |
| | | pT7_p3 | 44 | _insrev | EC130rev | TAAggtctcActtaGAACCCCTTTAAGCTTAGATCAAAGTC | |
| pP5_EspP | pSNAP-Esp2 | | - | - | EC1fwd | - | Type IIS |
| | pSNAP-Esp2 | | - | - | EC1rev | - | |
| | | pT7_p5 | 45 | _insfwd | EC33fwd | AAAcacctgcATTAggccATGGAAAACACAAACATCGTAAAGG | |
| | | pT7_p5 | 46 | _insrev | EC33rev | TAAcacctgcATTAccagTAGGGATAGTTGTAAGCTAAAGAATACGTTACC | |
| | | oligos | - | - | EC5a | - | |
| | | oligos | - | - | EC5b3 | - | |
| pP5_EspP_delta | pP5_EspP | | 47 | _vecfwd | EC39fwd | AAAcacctgcATCAaggaAGAAACAGTTACTACTATTTTTGC | Type IIS |
| | pP5_EspP | | 48 | _vecrev | EC39rev | TAAcacctgcATATtcctCTTCTATAATTGTTCCATCTGGAAGG | |
| pP6_EspP | pSNAP-Esp2 | | - | - | EC1fwd | - | Type IIS |
| | pSNAP-Esp2 | | - | - | EC1rev | - | |
| | | pT7_p6 | 49 | _insfwd | EC3fwd | AAAACCTGCATATGGCCATGGCAAAAATGATGC | |
| | | pT7_p6 | 50 | _insrev | EC3rev | TAAACCTGCATATCCAGTTCAGCAACCTGTTCTTCTG | |
| | | oligos | - | - | EC5a | - | |
| | | oligos | - | - | EC5b3 | - | |
| pP6_EspP_mut | pP6_EspP | | 51 | _vecfwd | E179fwd | AAAggtctcAGAAATCACAgcggcaACCGTCAACGTTGCCAAAATGGtgatg | Type IIS |
| | pP6_EspP | | 52 | _vecrev | EC179rev | AAAggtctcAtttcggcCTGCATCATggcTGCCATGGCCTGCGCTACG | |
| pP2_EspP | pSNAP-Esp2 | | - | - | EC1fwd | - | Type IIS |
| | pSNAP-Esp2 | | - | - | EC1rev | - | |
| | | pET23d_p2_synthetic | 53 | _insfwd | EC2fwd | AAACACCTGCATTAGGCCATGAAACACATGCCTC | |
| | | pET23d_p2_synthetic | 54 | _insrev | EC2rev | TAACACCTGCATATCCAGTTTGATCGTAAAGGTATCATCAAC | |
| | | oligos | - | - | EC5a | - | |
| | | oligos | - | - | EC5b3 | - | |
| **Ligase** | *T4DL was inserted into the EspP and LO vectors, a mutant (K159G) was constructed, and AMSI/IMSI barcodes were added to each.* | | | | | | |
| pT4_EspP | pSNAP_EspP2 | | 55 | _vecfwd | EC146fwd | TTCATGAGGTAACTGGTCTACTGGCGGAAGCAGCG | Gibson |
| | pSNAP_EspP2 | | 56 | _vecrev | EC146rev | TTCAGAATTTTAAGAATCATGGCCTGCGCTACGG | |
| | | pVP01_T4DNAligase | 57 | _insfwd | EC147fwd | ATGATTCTTAAAATTCTGAACGAAATAGCATC | |
| | | pVP01_T4DNAligase | 58 | _insrev | EC147rev | TAGACCAGTTACCTCATGAAAATCACC | |
| | | oligos | 59 | _ins2fwd | EC5a | - | |
| | | oligos | 60 | _ins2rev | EC5b3 | - | |
| pLOT4 | pLO_SNAP | | 61 | _vecfwd | EC148fwd | TTCATGAGGTAACTGGTCTAtaagCTTGACCTGTGAAGTGAAAAATG | Gibson |
| | pLO_SNAP | | 62 | _vecrev | EC148rev | TTCAGAATTTTAAGAATCATtgatccggtcgcaatttcc | |
| | | pVP01_T4DNAligase | - | - | EC147fwd | - | |
| | | pVP01_T4DNAligase | - | - | EC147rev | - | |
| pLOT4-K159G_ims | pLOT4_ims (intermediate) | | 63 | _vecfwd | EC166fwd | AAAcacctgcATTAggtGCTGATGGAGCTCGGTG | Type IIS |
| | pLOT4_ims (intermediate) | | 64 | _vecrev | EC166rev | TAAcacctgcATATcaccTAACTGAGCAAAGGCTGGAAATTTG | |

**Table S2.**

| Oligonucleotides for Binding Assays and Ligation Assays | |
|---|---|
| **Oligonucleotide name** | **Sequence** |
| | |
| **DNA Binding** | |
| *cholesterol-based* | |
| EC98a_unmod | GGA AGA GCA CCG ACC AGA ATC ATG CAA GTG CGT AAG ATA GTC GCG GGC CGG GCT CGG ATC |
| EC98b_FAM_chol | GAT CCG AGC CCG GCC CGC GAC TAT C/iFluorT/T ACG CAC TTG CAT GAT TCT GGT CGG TGC TCT TCC /3CholTE[illegible] |
| EC98b_unmodified | GATCCGAGCCCGGCCCGCGACTATCTTACGCACTTGCATGATTCTGGTCGGTgctcttcC |
| | |
| **RNA Binding** | |
| EC184_fwd_RNA_TYE | /5TYE665/UUCUGCGCGUAAUCUGCUGCUUG |
| EC184_rev_RNA_chol | CAAGCAGCAGAUUACGCGCAGAA-CholTEG |
| EC184_fwd_DNA_FAM | TTCTGCGCG/ifluorT/AATCTGCTGCTTG |
| EC184_rev_DNA_chol | CAAGCAGCAGATTACGCGCAGAA/3CholTEG/ |
| | |
| **Ligase assay substrates** | |
| *solution substrates* | |
| MR9 | /5'FAM/AACAGGATTAGCAG |
| MR10-f1 | /5Phos/AGCGAGGTATGTAGGCGGTG/3Cy5Sp/ |
| MR11-54PS | A*C*G*TGGCTAAGTGCTTATCTCACCGCCTACATACCTCGCTCTGCTAATCCTGTT |
| | |
| *on cell surface* | |
| EC164a_ROX / LIG_acceptor1 | /56-ROXN/CACCGCCTACATACCTCGCT |
| EC164b / LIG_donor1, 5'P | /5Phos/CTGCTAATCCTGTT |
| EC164c_FAM / LIG_acceptor2, 5'biotin | /56-FAM/AACAGGATTAGCAGAGCGA |
| EC164d_chol / LIG_donor2, 5'P, 3'chol | /5Phos/GGTATGTAGGCGGTGtttttttttttttttttttt/3CholTEG/ |
| | |
| **Ligase capture** | |
| EC164a / LIG_acceptor1 | CACCGCCTACATACCTCGCT |
| EC164b / LIG_donor1, 5'P | /5Phos/CTGCTAATCCTGTT |
| EC164c / LIG_acceptor2, 5'biotin | /5BiotinTEG/AACAGGATTAGCAGAGCGA |
| EC164d / LIG_donor2, 5'P, 3'chol | /5Phos/GGTATGTAGGCGGTGtttttttttttttttttttt/3CholTEG/ |

| Oligonucleotides for Model Selection Diagnostic PCRs | |
|---|---|
| **Primer name** | **Sequence** |
| EC19 | GGAACCGCTGATGCAGG |
| EC20 | CGCCAGCTGCTGATAGC |

**Table S3.** **Plasmids and gBlocks**

| Plasmids | Provenance | Reference |
| --- | --- | --- |
| pNGAL97 | Arne Skerra, Technische Universität München | Binder et al., 2010 |
| pSNAP-tag(T7_2) | NEB | N9181S |
| pET23d_p2(N62D) | Leticia Torres | Torres and Pinheiro, 2018 |
| pT7_P3 | Salas group, Autonomous University of Madrid | Mencía et al., 2012 |
| pT7_P5 | Salas group, Autonomous University of Madrid | Mencía et al., 2012 |
| pT7_P6 | Salas group, Autonomous University of Madrid | Mencía et al., 2012 |
| pET-23d(+) | Novagen (Sigma) | 69748 |
| pVP01_T4DNAligase | Marleen Renders and Vitor Pinheiro | This study |

**Table S4.** **Strains**

| Abbreviated name | Full name | Source | Product Code | Link if available |
|---|---|---|---|---|
| DH10B | DH10-beta | NEB | C3019I | https://www.neb.com/products/c3019-neb-10-beta-competent-e-coli-high-efficiency#Product%20Information |
| T7 | T7 Express *lysY* /Iq | NEB | C3013I | https://www.neb.com/products/c3013-t7-express-lysyiq-competent-e-coli-high-efficiency#Product%20Information |
| C41 | C41(DE3) | Renos Savva group / Lucigen | 60341 | https://www.lucigen.com/OverExpress-C41-DE3-and-C43-DE3-Competent-Cells/ |
| dsbA | BW25113_*dsbA* - | Filipe Cabreiro group / Keio collectior | OEC4988Unit | https://dharmacon.horizondiscovery.com/cdnas-and-orfs/non-mammalian-cdnas-and-orfs/e-coli/e-coli-keio-knockouts/ |
| DH10B(*ompT* -) | DH10B(*ompT* -) | Valerie Pezo / Philipe Marliere | n/a | n/a |

**Table S5. Gene sequences**

**LppOmpA**

CCCAATCTAGAAAGGAGATATACACTTCATGAAAGCGACCAAACTGGTGCTGGGCGCGG
TGATTCTGGGCAGCACCCTGCTGGCGGGCTGCAGCAGCAACGCGAAAATTGATCAGAAC
AACAACGGCCCGACCCATGAAAACCAGCTGGGCGCGGGCGCGTTTGGCGGCTATCAGGT
GAACCCGTATGTGGGCTTTGAAATGGGCTATGATTGGCTGGGCCGCATGCCGTATAAAG
GCAGCGTGGAAAACGGCGCGTATAAAGCGCAGGGCGTGCAGCTGACCGCGAAACTGGG
CTATCCGATTACCGATGATCTGGATATTTATACCCGCCTGGGCGGCATGGTGTGGCGCGC
GGATACCAAAAGCAACGTGTATGGCAAAAACCATGATACCGGCGTGAGCCCGGTGTTTG
CGGGCGGCGTGGAATATGCGATTACCCCGGAAATTGCGACCGGATGATGAGACCTTT

**p16.7**

TTTAACTTTAAGAAGGAGATATACCATGGAAGCTATTTTAATGATCGGTGTACTTGCATT
GTGCGTTATATTCCTTCTATCAGGACGAAACAACAAAAAGAAACAGGAAGCAAGGGAGC
TAGAAGATTATCTTGAAGACCTCAACAAACGAGTTGTTCAACGAACACAAATACTCAGC
GAGCTTAACGAAGTTATCTCAAACAGAAGCATTGACAAAACAGTCAACCTGTCAGCTTG
TGAAGTCGCCGTGCTTGATCTGTATGAGCAGTCAAATATCCGCATTCCTAGTGACATCAT
CGAAGATTTGGTTAATCAACGTTTACAAAGTGAACAGGAAGTGTTAAACTATATAGAGA
CACAGCGGACATACTGGAAATTGGAGAATCAGAAAAAACTATATCGGGGGTCATTGAAA
TGAGGCCGCACTCGAGCACCACCACCAC

**INP**

ATGACTCTCGACAAGGCGTTGGTGCTGCGTACCTGTGCAAATAACATGGCCGATCACTGC
GGCCTTATATGGCCCGCGTCCGGCACGGTGGAATCCAGATACTGGCAGTCAACCAGGCG
GCATGAGAATGGTCTGGTCGGTTTACTGTGGGGCGCTGGAACCAGCGCTTTTCTAAGCGT
GCACGCCGATGCTCGATGGATTGTCTGTGAAGTTGCCGTTGCAGACATCATCAGTCTGGA
GGAGCCGGGAATGGTCAAGTTTCCGCGGGCCGAGGTGGTTCATGTCGGCGACAGGATCA
GCGCGTCACACTTCATTTCGGCACGTCAGGCCGACCCTGCGTCAACGTCAACGTCAACGT
CAACGTCAACGTTAACGCCAATGCCTACGGCCATACCCACGCCCATGCCTGCGGTAGCA
AGTGTCACGTTACCGGTGGCCGAACAGGCCCGTCATGAAGTGTTCGATGTCGCGTCGGTC
AGCGCGGCTGCCGCCCCAGTAAACACCCTGCCGGTGACGACGCCGCAGAATTTGCAGAC
CGCCACTTACGGCAGCACGTTGAGTGGCGACAATCACAGTCGTCTGATTGCCGGTTATGG
CAGTAACGAAACCGCTGGCAACCACAGTGATCTAATTGGATCTGGCGGGCATGACTGCA
CCCTGATGGCGGGAGATCAAAGCAGATTGACCGCTGGTAAGAACAGTGTCTTGACGGCA
GGCGCTCGTAGCAAACTTATTGGCAGTGAAGGCTCGACGCTCTCGGCTGGAGAAGACTC
CACACTAATTTTCAGACTCTGGGACGGGAAGAGGTACAGGCAACTGGTCGCCAGAACGG
GTGAGAACGGTGTTGAGGCCGACATACCGTATTACGTGAACGAAGATGACGATATTGTC
GATAAACCCGACGAGGACGATGACTGGATAGAGGTAAAGCCCGGATCTGCAGAAGCTGC
AGCTAAAGAAGCAGCTGCAAAGGAAGCTGCAGCTAAAGAAGCAGCTGCAAAGGCCATG
GCAGAAGCGGTCTGA
